# Tetrahydrocannabinol (THC) bei Patienten mit Fibromyalgiesyndrom (FMS): Retrospektive Untersuchung zu Veränderungen von Schmerz, psychometrischen Variablen und Analgetikaverbrauch im Verlauf einer interdisziplinären multimodalen stationären Schmerztherapie (IMST)

**DOI:** 10.1007/s00482-023-00727-4

**Published:** 2023-06-08

**Authors:** Horst Bettstetter, Arne Schäfer

**Affiliations:** 1 Schmerzzentrum Inn-Salzach, Wackerstr. 7, 84489 Burghausen, Deutschland; 2 Fachbereich Psychodiabetologie, Diabetes-Klinik Bad Mergentheim, Theodor-Klotzbücher-Str. 12, 97980 Bad Mergentheim, Deutschland; 3 Medizinische Klinik und Poliklinik II, Universitätsklinikum Würzburg, Oberdürrbacherstr. 6, Haus A3, 97080 Würzburg, Deutschland

**Keywords:** Medizinisches Cannabis, Schmerzintensität, Depression, Lebensqualität, Analgetikaverbrauch, Medical cannabis, Pain intensity, Depression, Quality of life, Analgesic consumption

## Abstract

**Hintergrund:**

Seit dem 1. März 2017 ist medizinisches Cannabis (MC) bundesweit verschreibungsfähig. Zur Wirksamkeit von MC bei Fibromyalgiesyndrom (FMS) existieren bisher einige qualitativ unterschiedliche Studien.

**Fragestellung:**

Ziel der Untersuchung war, die Wirksamkeit von Tetrahydrocannabinol (THC) im Verlauf einer IMST hinsichtlich des Schmerzes und mehrerer psychometrischer Variablen zu untersuchen.

**Material und Methoden:**

Für die Studie wurden im Studienzeitraum (2017–2018) alle Patienten einer Schmerzstation, die an einem FMS erkrankt waren und in einem multimodalen, interdisziplinären Setting behandelt wurden, entsprechend den Einschlusskriterien selektioniert. Die Patienten wurden getrennt nach Gruppen mit und ohne THC-Medikation bzgl. der Schmerzintensität, verschiedener psychometrischer Parameter und des Analgetikaverbrauchs während des Aufenthalts untersucht.

**Ergebnisse:**

Von den 120 in die Studie eingeschlossenen FMS-Patienten wurden 62 Patienten (51,7 %) mit THC behandelt. In den Parametern Schmerzintensität, Depressivität und Lebensqualität zeigte sich eine signifikante Besserung der Gesamtgruppe während des Aufenthalts (*p* < 0,001), die durch den Einsatz von THC signifikant stärker ausfiel. In fünf der sieben untersuchten Analgetikagruppen konnte bei den mit THC behandelten Patienten signifikant häufiger die Dosis reduziert bzw. das Medikament abgesetzt werden.

**Schlussfolgerung:**

Die Ergebnisse liefern Hinweise dafür, dass THC als medikamentöse Alternative in Ergänzung zu den bisher in verschiedenen Leitlinien empfohlenen Substanzen für die Therapie des FMS in Betracht gezogen werden kann.

## Abstract (en)

**Background:**

Since March 1, 2017, medical cannabis (MC) can be prescribed nationwide in Germany. To date, there have been a number of qualitatively different studies on the effectiveness of MC in fibromyalgia syndrome (FMS).

**Objective:**

The aim of the study was to investigate the effectiveness of THC in the course of interdisciplinary multimodal pain therapy (IMPT) on pain and several psychometric variables.

**Materials and methods:**

For the study, in the period 2017–2018, all patients in the pain ward of a clinic who were suffering from FMS and were treated in a multimodal interdisciplinary setting were selected based on inclusion criteria. The patients were examined separately according to groups with and without THC about pain intensity, various psychometric parameters and analgesic consumption during the stay.

**Results:**

Of the 120 FMS patients included in the study, 62 patients (51.7%) were treated with THC. In the parameters of pain intensity, depression, and quality of life, there was a significant improvement in the entire group during the stay (*p* < 0.001), which was significantly greater through the use of THC. In five of the seven analgesic groups examined, the dose was reduced or the drug discontinued significantly more often in the patients treated with THC.

**Conclusion:**

The results provide indications that THC can be considered as a medical alternative in addition to the substances previously recommended in various guidelines.

**Graphic abstract:**

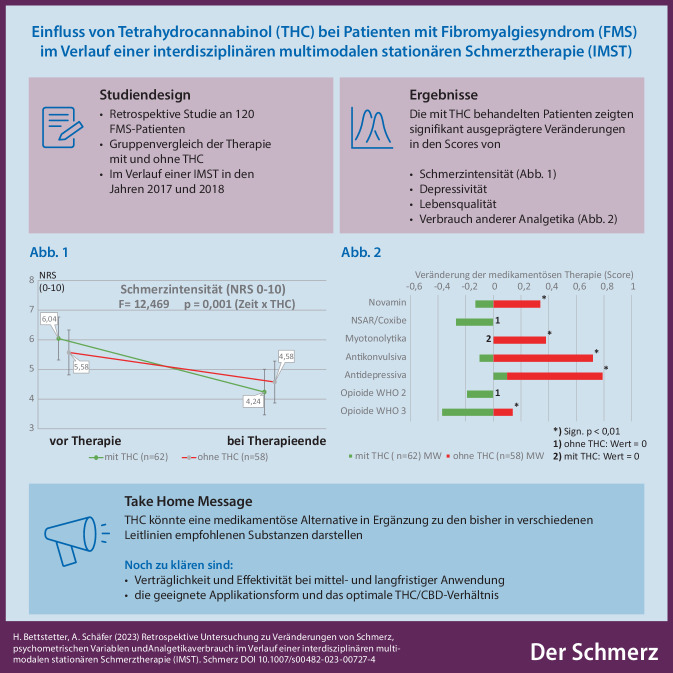

## Einführung

Das Fibromyalgiesyndrom (FMS) ist charakterisiert durch diffuse muskuloskeletale Schmerzen mit Fatigue, Störungen des Schlafs, kognitiven Defiziten und häufig weiteren psychosomatischen Symptomen. Die Prävalenz liegt weltweit bei 2,7 % [18].

Nach aktuellen Schmerzkonzepten gehört der Schmerz bei FMS zur Kategorie der noziplastischen Schmerzen [10]. Diese wurde als dritte Schmerzkategorie kürzlich zu den bisher bekannten nozizeptiven und neuropathischen Schmerzarten in die Terminologie der IASP übernommen [8].

Der in vielen Ländern in den letzten Jahren vereinfachte Zugang zur Verschreibung von cannabishaltigen Präparaten [1, 11] führte mangels Alternativen zu einem breiteren Einsatz insbesondere bei noziplastischen Schmerzzuständen.

Seit dem 1. März 2017 ist medizinisches Cannabis (MC) in Deutschland verschreibungsfähig und kann von Ärztinnen und Ärzten jeder Fachrichtung verordnet werden.

Die beiden relevanten chemischen Substanzen von medizinischem Cannabis (MC) sind Tetrahydrocannabinol (THC) und Cannabidiol (CBD; [13]). Beide haben sowohl einen Effekt auf den CB1-Rezeptor als auch auf den CB2-Rezeptor, THC als partieller Rezeptoragonist, CBD als negativer allosterischer Modulator des CB1-Rezeptors [7]. THC ist die zentral wirksame Substanz mit analgetischen, appetitanregenden und stimmungsstabilisierenden Effekten, während das nicht psychotrope CBD antiinflammatorische, anxiolytische und auch schmerzlindernde Wirkungen aufweist [24].

In einem aktuellen systematischen Literatur-Review zu MC bei FMS ergaben sich 22 relevante Artikel, darunter 2 randomisierte klinische Studien und 2 observationelle, prospektive Studien, die positive Effekte bei geringer Nebenwirkungsrate aufzeigten [9].

Die Wirksamkeit von MC im Verlauf einer IMST ist bislang nicht systematisch untersucht. Ziel der vorgestellten retrospektiven Studie war es deshalb, den Effekt von THC als dem Hauptwirkstoff von MC auf Schmerz und mehrere psychometrische Variablen zu untersuchen.

## Methoden

### Patientenkollektiv

Für die hier vorgestellte Untersuchung wurden die Datensätze aller Patienten mit der Diagnose FMS (ICD 79.70) der Schmerzabteilung unserer Klinik, die in den Jahren 2017 und 2018 an einer interdisziplinären multimodalen stationären Schmerztherapie (IMST) teilgenommen hatten, retrospektiv ausgewertet. Hierfür wurde von den Patienten eine schriftliche Einverständniserklärung eingeholt. Die Studie wurde vom Ethikkomitee des Klinikverbunds gebilligt und in Übereinstimmung mit den Forschungsvorschriften, den geltenden internationalen GCP-Standards sowie den in der Deklaration von Helsinki dargelegten ethischen Grundsätzen durchgeführt.

Die Indikation für die stationäre Therapie wurde gemäß den Vorgaben im OPS in einem ärztlichen Assessment vor der stationären Aufnahme gestellt. Dabei wurde überprüft, ob mindestens drei der folgenden Kriterien erfüllt waren:

- Vorherige erfolglose ambulante Therapie
- Starke Beeinträchtigung der Lebensqualität mit Störung der Lebensführung oder Berufstätigkeit
- Inadäquate medikamentöse Therapie
- Psychosoziale Risikofaktoren bzw. psychische Komorbidität
- Relevante somatische Komorbidität

Die Aufenthaltsdauer betrug gemäß den Vorgaben des OPS [DIMDI 2016^1^] entweder mindestens 7 bis maximal 13 Tage (Mittelwert 9,2 Tage – OPS 8‑918.0) oder mindestens 14 bis maximal 20 Tage (Mittelwert 16,7 Tage – OPS 8‑918.1).

Zu Beginn der Behandlung wurden die Patienten von einem ärztlichen Schmerztherapeuten, einem Psychotherapeuten und einem Physiotherapeuten untersucht und das Programm nach Bedarf individuell angepasst. Die Diagnose eines FMS wurde durch den ärztlichen Schmerztherapeuten aufgrund der ACR-EULAR-Kriterien von 2010 gestellt. Das Chronifizierungsstadium nach Gerbershagen [17] wurde zu Beginn des Aufenthalts bestimmt und im Arztbrief ausgewiesen.

Das strukturierte therapeutische Programm umfasste Einzel- und Gruppentherapien mit maximal 8 Teilnehmern aus den Bereichen Edukation, Physiotherapie, medizinische Trainingstherapie, übende psychologische und körperliche Verfahren, Kunsttherapie, Naturheilverfahren, Wassergymnastik und medikamentöse Einstellung. Einmal pro Woche fanden interdisziplinäre Teamsitzungen statt, in denen das therapeutische Prozedere angepasst wurde. Jeder Patient bekam vor der stationären Aufnahme den Deutschen Schmerzfragebogen und vor Entlassung einen Verlaufsfragebogen zum Ausfüllen.

Nachdem ab März 2017 cannabisbasierte Medikamente (z. B. Dronabinol) bzw. medizinisches Cannabis (z. B. Blüten) als Kassenleistung verordenbar waren, wurden sie bei Patienten mit FMS, die keine ausreichende Schmerzreduktion durch die Leitlinientherapie [23] erfahren hatten, als weitere medikamentöse Therapieoption in Betracht gezogen. Als Substanz wurde in dem untersuchten Zeitraum ausschließlich Dronabinol, das psychoaktive Stereoisomer des THC ((−)-∆^9^-*trans*-Tetrahydrocannabinol), verwendet [4]. Als Darreichungsform kam überwiegend die Kapselform zum Einsatz (nur 5 Fälle mit Einnahme in flüssiger Form). Nach Indikationsstellung wurde in der Regel mit Kapseln von 1,25 mg begonnen und die Dosis pro Tag um 1,25 bis 2,5 mg gesteigert, beim Einsatz von Tropfen mit 2 Tropfen (1,6 mg) begonnen und tgl. um 1–2 Tropfen erhöht. Beim Auftreten von Nebenwirkungen wurde erst wieder erhöht, wenn diese abgeklungen waren. Endpunkte der Dosiserhöhungen waren eine für den Patienten suffiziente Schmerzreduktion, intolerable Nebenwirkungen oder ein Erreichen einer Maximaldosis von 33 mg, was der gesetzlichen Verschreibungshöchstmenge für Cannabisvollextrakte entspricht.

Um den klinischen Einfluss einer Dronabinolmedikation während einer IMST zu untersuchen, wurden Patienten mit und ohne Dronabinolmedikation bzgl. des Parameters Schmerzstärke und einiger psychologischer Variablen des Verlaufsfragebogens miteinander verglichen.

### Messverfahren und Auswertungsdesign

Die erhaltenen 152 Datensätze wurden auf folgende Ausschlusskriterien hin überprüft (in Klammern: Anzahl der Ausschlüsse): Alter < 18 Jahre, schriftliches Einverständnis für die Studienteilnahme, unvollständige Datensätze (*n* = 25), laufende THC-Therapie bei Aufnahme (*n* = 1), Abbruch einer während des Aufenthalts begonnenen THC-Therapie wegen Nebenwirkungen (*n* = 2), vorzeitige Beendigung der stationären Schmerztherapie (*n* = 4). Von diesen 4 Patienten beendeten 2 die Schmerztherapie aus persönlichen Gründen, 2 wurden nach Auftreten von internistischen Erkrankungen von der Schmerzstation verlegt. Zur endgültigen Auswertung kamen 120 Datensätze. Die Auswertung der Fragebögen erfolgte EDV-basiert. Die folgenden psychometrischen Daten wurden als abhängige Variablen im Längsschnitt und im Gruppenvergleich mit und ohne THC-Medikation erhoben: Depressivität (HADS‑D [6]: Werte 0–21, 0–7 unauffällig, 8–10 grenzwertig, 11–21 auffällig), Angst (HADS‑A [6]: Werte 0–21, 0–7 unauffällig, 8–10 grenzwertig, 11–21 auffällig), Lebensqualität (QLIP [16]: Werte 0–43, kein Cut-off) und Schlafstörung (PDI Schlaf [16]: Werte 0–10). Zur Beurteilung des Verlaufs der Schmerzintensität wurden die ersten und letzten drei von der Pflegekraft erhobenen NRS-Werte (11-stufige Skala von 0 bis 10) verwendet.

Zur Beurteilung der Veränderung der Medikation während des stationären Aufenthalts wurde für die verschiedenen Analgetikagruppen jeweils ein einheitlicher Score verwendet:

−2: Absetzen des Medikaments
−1: Dosisverringerung
- 0: nicht verordnet oder keine Dosisveränderung während des Aufenthalts
- +1: Dosissteigerung
- +2: neue Verordnung des Medikaments

Innerhalb der beiden Patientengruppen wurde für jede Analgetikagruppe zum Zwecke der besseren Übersichtlichkeit und der weiteren Datenverarbeitung ein arithmetischer Mittelwert der Scores gebildet.

### Statistik

Die Datenerfassung und -analyse erfolgte mithilfe des SPSS-Softwarepakets (SPSS für Windows, deutschsprachige Version 17.0.0, SPSS-Inc., Chicago, IL, USA). Entsprechend dem verwendeten Studiendesign (retrospektiv, zweifaktoriell mit einer abhängigen [Zeit] und einer unabhängigen Variablen [THC-Medikation]) erfolgte die zentrale inferenzstatistische Auswertung mithilfe des allgemeinen linearen Modells (zweifaktorielle Varianzanalyse mit Messwiederholung). Für einfache Mittelwertsvergleiche ohne die Einbeziehung zusätzlicher Variablen kamen entsprechend t‑Tests für abhängige (Zeitverlauf) oder unabhängige (Querschnitt) Stichproben zum Einsatz.

Für die Beschreibung der Stichprobe und die sonstige beschreibende Darstellung der gewonnenen Daten und deren Verteilung wurden die üblichen Parameter verwendet (z. B. das arithmetische Mittel als Lagemaß und die Standardabweichung als Streumaß bei intervallskalierten Daten). Für ordinal- oder nominalskalierte Daten wurden die zugehörigen absoluten bzw. relativen Häufigkeiten („Prozentsätze“) angegeben.

Zum gewählten Stichprobenumfang ist zu bemerken, dass im Vorfeld keine Berechnungen zur Bestimmung des optimalen Stichprobenumfangs angestellt wurden, da es sich um eine retrospektive Datenanalyse handelt. Es wurde dennoch darauf geachtet, dass mithilfe der vorgenommenen Auswertungen zumindest auch mittelgroße Effekte detektiert werden konnten.

## Ergebnisse

Von den 58 nach Standard behandelten Patienten (Vergleichsgruppe) waren 55 (95 %) weiblich und 3 (5 %) männlich, in der Dronabinolgruppe (*N* = 62) waren 51 (82 %) weiblich und 11 (18 %) männlich (signifikanter Gruppenunterschied, *p* < 0,045). In der THC-Gruppe befanden sich signifikant mehr Patienten im Chronifizierungsstadium 3 (93,5 %) als in der Vergleichsgruppe (77,6 %; Chi-Quadrat-Test, *p* < 0,012 [keine Patienten im Chronifizierungsstadium 1]). In der Dronabinolgruppe blieben 27 (44 %) kürzer als 14 Tage (OPS 8‑918.0), in der Vergleichsgruppe 15 (26 %, nichtsignifikanter Unterschied).

Weitere Daten zu Demografie, Krankheitsdauer und Therapieintensität sind in Tab. 1 aufgeführt.

**Tab. 1:** Demografie, Krankheitsdauer und Therapieintensität

Gruppen	Dronabinol (*N* = 62)		Standardtherapie (*N* = 58)		
	MW	SD	MW	SD	Signifikanz
*Alter*	53,7	9,1	55,8	9,6	NS
*BMI*	29,5	6,2	30,5	7,1	NS
*Krankheitsdauer in Jahren*	7,5	6,9	7,0	6,1	NS
*Aufenthaltsdauer (Tage)*	13,5	3,8	14,7	3,4	NS
*Therapieanzahl gesamt*	73,6	19,9	75,6	17,9	NS
Mit physiotherapeutischen Anteilen	43,0	11,7	45,4	12,0	NS
Mit psychotherapeutischen Anteilen	24,8	8,5	24,3	6,6	NS

*N* Stichprobenumfang, *MW* Mittelwert, *SD* Standardabweichung, *NS* nicht signifikant

Die zumeist hoch chronifizierten Patienten hatten neben FMS noch eine Anzahl weiterer Schmerzdiagnosen, die nach Standard therapierten Patienten im Mittel 3,3 (SD = 1,4), die mit Dronabinol behandelten 3,7 (SD = 1,6; nichtsignifikanter Unterschied). Die prozentuale Verteilung der häufigsten Schmerzdiagnosen ist in Tab. 2 nach Gruppen aufgeführt.

**Tab. 2:** Prozentuale Verteilung der häufigsten Schmerzdiagnosegruppen

Schmerzdiagnosen	Dronabinol (*N* = 62)	Standardtherapie (*N* = 58)	
	Anteil (%)	Anteil (%)	Signifikanz
Schmerzsyndrom der Wirbelsäule	87,1	89,7	NS
Schmerzsyndrom der Extremitäten	67,7	70,7	NS
Neuropathisches Schmerzsyndrom	46,8	34,5	NS
Kopfschmerzsyndrom	50,0	22,4	*p* < 0,002
Entzündliche rheumatologische Erkrankung	14,5	10,3	NS

*N* Stichprobenumfang, *NS* nicht signifikant, *p* Irrtumswahrscheinlichkeit

Die Patienten wiesen in der Dronabinolgruppe im Durchschnitt 1,6 (SD = 1,3) und in der Vergleichsgruppe 1,4 (SD = 1,1; nichtsignifikanter Unterschied) psychische Diagnosen auf. Die prozentuale Verteilung der häufigsten psychischen Diagnosegruppen ist in Tab. 3 aufgeführt.

**Tab. 3:** Prozentuale Verteilung der häufigsten psychologischen Diagnosegruppen

Psychische Diagnosen	Dronabinol (*N* = 62)	Standardtherapie (*N* = 58)	
	Anteil (%)	Anteil (%)	Signifikanz
Depression	82,3	86,2	NS
Angstsyndrom	27,4	19,0	NS
Posttraumatische Belastungsstörung	12,9	8,6	NS
Persönlichkeitsstörung	11,3	3,4	NS
Somatoforme Störung	16,1	13,8	NS
Suchterkrankung	9,7	3,4	NS

*N* Stichprobenumfang, *NS* nicht signifikant

Die durchschnittliche Anzahl der somatischen Komorbiditäten betrug in der Dronabinolgruppe 3,3 (SD = 1,9), in der Vergleichsgruppe 2,4 (SD = 1,7, signifikanter Unterschied mit *p* < 0,005).

Die medikamentöse Vortherapie wurde bzgl. der Vorgaben der FMS-Leitlinie [23] aufgrund der anamnestischen Daten erfasst. Dabei war der Anteil der voll ausgeschöpften medikamentösen Therapie in der THC-Gruppe mit 83,9 % signifikant höher als in der Kontrollgruppe (32,8 %; *p* < 0,001).

Die Aufdosierung mit Dronabinol verlief im Schnitt über 13,4 Tage (SD 3,68); dabei wurden 14,8 mg (SD = 6,02) als durchschnittliche THC-Dosis verabreicht (min. 2,6 mg, max. 31,3 mg).

**Abb. 1:**
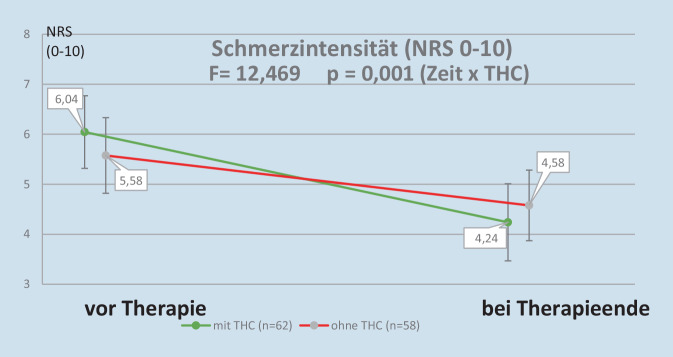
Veränderung der Schmerzintensität während der IMST

Die mit Dronabinol behandelten Patienten zeigten im Vergleich zu den nach Standard therapierten Patienten eine signifikant stärkere Schmerzreduktion während des stationären Aufenthalts (vgl. Abb. 1). Obwohl der Ausgangswert auf der 11-stufigen Skala von 0 bis 10 bei den mit THC behandelten Patienten um 0,5 Punkte höher lag, erreichte der Wert nach 2 Wochen einen um 0,3 Einheiten niedrigeren Wert.

**Abb. 2:**
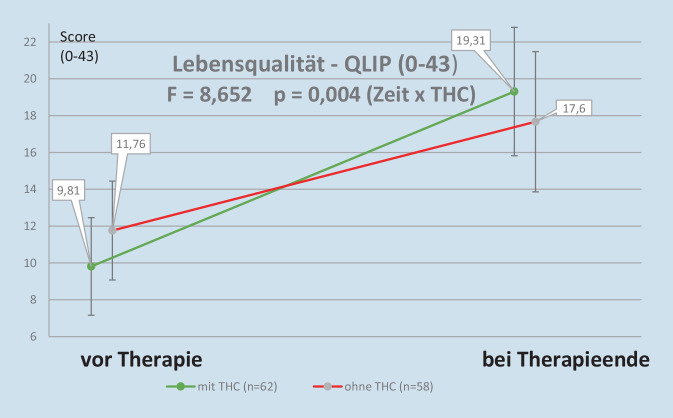
Veränderung der Lebensqualität während der IMST

Ähnlich kam es bei der grafischen Darstellung des Verlaufs der Lebensqualität (QLIP) zu einer Überkreuzung der den beiden Gruppen zugeordneten Linien, da bei den THC-Patienten trotz stärkerer Beeinträchtigung zu Beginn der Therapie ein höherer Endwert erreicht wurde (vgl. Abb. 2).

**Abb. 3:**
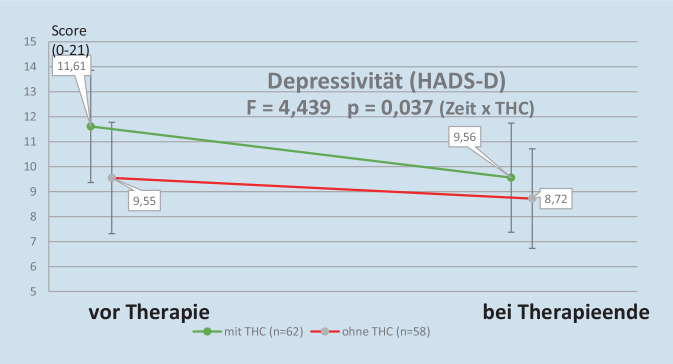
Veränderung des Depressivitätswerts während der IMST

Die Depressivitätsscores (gemessen als HADS-D) veränderten sich im Vergleich dazu etwas geringer und nicht so deutlich unterschiedlich zwischen den beiden betrachteten Patientengruppen, sodass es zu keiner Überkreuzung der Grafiklinien kam. Dies spiegelt sich auch im niedrigeren Signifikanzniveau von 0,037 wider (vgl. Abb. 3). Dennoch profitiert auch hier die Patientengruppe mit THC signifikant stärker mit Blick auf die Reduktion des Depressivitätsscores (signifikanter Wechselwirkungseffekt Zeit × Gruppe).

Keine Gruppenunterschiede ergaben sich bei den Werten des Angstscores (HADS‑A; Dronabinolgruppe vs. Vergleichsgruppe: vorher 11,23 ± 4,77 vs. 10,21 ± 4,88; nachher 9,56 ± 4,37 vs. 8,72 ± 3,98) und des PDI Schlaf (Dronabinolgruppe vs. Vergleichsgruppe: vorher 7,13 ± 2,19 vs. 6,14 ± 2,45; nachher 5,52 ± 2,94 vs. 5,02 ± 2,45).

**Abb. 4:**
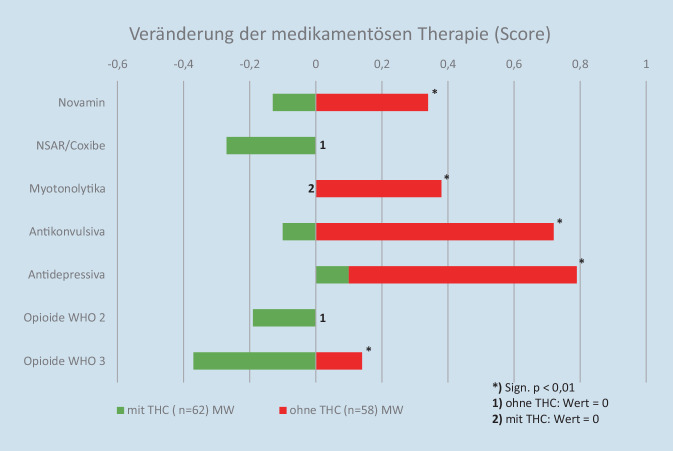
Veränderung der Medikation, aufgeschlüsselt nach Analgetikagruppen

Die Veränderung der Medikation während des stationären Aufenthalts wurde aufgeschlüsselt nach Analgetikagruppen ausgewertet. In fünf der sieben untersuchten Analgetikagruppen konnte bei den mit Dronabinol behandelten Patienten signifikant häufiger die Dosis reduziert bzw. das Medikament abgesetzt werden (vgl. Abb. 4).

## Diskussion

Habib u. Artul (2018; [5]) beschreiben die Veränderungen durch die MC-Therapie bei einigen FMS-Patienten als dramatisch. Der Autor hat ähnlich eklatante Verbesserungen in seiner Tätigkeit als Rheumatologe vorher nur nach dem Einsatz von Kortikoiden bei entzündlich-rheumatischen Erkrankungen gesehen. Ähnlich erging es uns nach dem Einsatz von THC bei unseren FMS-Patienten. So war dies der Anlass dafür, sich mit dieser Thematik intensiver auseinanderzusetzen und führte letztlich zu dieser retrospektiven Studie.

In unserer Untersuchung wurde ausschließlich Dronabinol verwendet, zu über 90 % in Kapselform und zu einem geringen Teil als ölige Lösung in Tropfenform. Durch das stationäre Setting mit täglichen Visiten war eine enge ärztliche Führung während der Aufdosierungs- und initialen Erhaltungsphase gewährleistet. Eine vorsichtige Eindosierung ist unserer Erfahrung nach bei FMS-Patienten wegen der besseren Verträglichkeit essenziell. Auch von den Autoren MacCallum et al. (2018; [12]) wird eine langsame Titration nach dem Motto „start low, go slow, and stay low“ mit einer maximalen Dosierung von 30 mg THC empfohlen.

In einem 2021 erschienenen Review [9] werden im Zeitraum von 2019 bis 2021 sieben vergleichbare Studien aufgeführt. Unsere Ergebnisse werden im Folgenden im Wesentlichen diesen Arbeiten und einigen weiteren Arbeiten gegenübergestellt.

In unserer Untersuchung lag die durchschnittliche Dosis von THC am Ende der Auftitrierungsphase bei 14,75 mg. Die niedrigste orale THC-Dosis bei ähnlichen Studien lag bei 4,4 mg [2], die höchste bei 46,2 mg [14]. Bei inhalativer Applikation betrug sie ca. 20–50 mg [5, 20, 27].

Es gibt wenige Studien zum Schmerzverlauf bei hospitalisierten FMS-Patienten, da die meisten interdisziplinären Therapien international im ambulanten Setting durchgeführt werden. Bei unserer Vergleichsgruppe reduzierte sich der gemittelte Schmerzwert von NRS 5,6 auf 4,6. Ähnlich veränderte sich der Schmerzwert bei FMS-Patienten des Imanuel Krankenhauses Berlin von 6,7 auf 5,8 bei einer Verweildauer von 14 Tagen [15]. Bei unseren mit THC behandelten Patienten verbesserte sich der NRS-Wert nach dem Beobachtungsintervall von zwei Wochen von 6,0 auf 4,2 um 1,8 Punkte. In einem ähnlichen Bereich lagen die Verbesserungen bei Studien, die Beobachtungszeiträume von bis zu einem Monat umfassten [19, 22], bei längeren Beobachtungsphasen lagen die Verbesserungen meist höher ([2, 26, 27], maximal mit 5,5 bei Habib nach 10 Monaten [5]). Zu keiner Besserung des Spontanschmerzes kam es bei van de Donk [25] nach einer einmaligen MC-Inhalation, jedoch zu einer Anhebung der Druckschmerzschwelle. Daraus könnte man folgern, dass die Reduktion der Schmerzintensität ein erst nach längerem Intervall auftretendes Phänomen darstellt. Ob eine Anhebung der Druckschmerzschwelle den neurophysiologischen Mechanismus für die Schmerzreduktion darstellt, muss noch geklärt werden. In einer Untersuchung von 2006 stellten Schley et al. [21] nach Einnahme von 10 bis 15 mg THC neben einer Abnahme des Spontanschmerzes eine Reduktion der Schmerzempfindung auf einen elektrischen Reiz fest.

In unserer Untersuchung kam es zu einer deutlichen Besserung der Lebensqualität bei Therapieende, die bei den mit THC behandelten Patienten signifikant stärker ausgeprägt war. In keiner vergleichbaren Studie wurde bisher der QLIP erfasst. Vergleichbare Lebensqualitätsparameter mit signifikanter Besserung finden sich bei Safakish [19], bei Chaves et al. [2] und bei Sagy et al. [20].

Die mit THC behandelten Patienten zeigten eine signifikant stärkere Abnahme des Depressivitätswerts (HADS-D). Vergleichbar waren die diesbezüglichen Ergebnisse bei Sagy et al. [20], Habib et al. [5] und bei Safakish et al. [19]. Im Gegensatz dazu kam es bei den Patienten von Mazza et al. [14] zu keiner signifikanten Verbesserung.

Bei der Messung des Angstwerts (HADS-A) ergab sich bei Mazza et al. [14] ähnlich wie bei unseren Patienten keine Verbesserung. Bei Habib et al. [5] dagegen besserte sich der Angstscore von 6,84 auf 2,42, bei Skrabek et al. [22] um 1,67 Punkte.

Die Schlafqualität zeigte bei unseren Patienten keine Verbesserung im Gegensatz zu Safakish et al. [19], Giorgi et al. [3], Sagy et al. [20] und Habib et al. [5]. Unser Ergebnis lässt sich evtl. dadurch erklären, dass die Patienten nicht in ihrer gewohnten Umgebung schliefen.

Die Veränderung der Medikation während des stationären Aufenthalts wurde in unserer Untersuchung aufgeschlüsselt nach Analgetikagruppen ausgewertet. In fünf der sieben untersuchten Gruppen konnte bei den mit THC behandelten Patienten signifikant häufiger die Dosis reduziert bzw. das Medikament abgesetzt werden – lediglich in den Gruppen „NSAR“ und „Opioide der WHO-Gruppe 2“ zeigte sich keine signifikante Reduktion.

Auch Yassin et al. [27] verwendeten einen Score zur Ermittlung der Medikamentenveränderung. Es zeigte sich auch bei ihm eine signifikante Reduktion des Scores, die mit der Zunahme der Beobachtungsdauer (6 vs. 3 Monate) stärker ausgeprägt war. Eine Reduktion bzw. ein Absetzen der weitern Analgetika findet sich auch bei Mazza et al. [14], bei Habib et al.[5], bei Giorgi et al.[3], bei Sagy et al. [20] und bei Safakish et al. [19].

Die Nebenwirkungen von MC sind abhängig von der Applikation (oral oder inhalativ) und dem Vorgehen bei der Dosisfindung. Die häufigsten Nebenwirkungen sind in den vergleichbaren Studien Müdigkeit bis Benommenheit, Schwindel, Mundtrockenheit, Palpitationen, Appetitzunahme, Unruhegefühle, bei inhalativer Therapie zusätzlich Augenrötungen und Halsschmerzen. Die Symptome sind umso ausgeprägter, je schneller die Aufdosierung durchgeführt wurde. Die höchste nebenwirkungsbedingte Ausfallrate von 48,6 % (meist aufgrund von Verwirrtheit) bei Mazza et al. ist vermutlich auf die hohe Dosis von 46,2 mg THC bei oraler Einnahme zurückzuführen. Weit geringere Ausfallraten aufgrund von Nebenwirkungen fanden sich bei van de Donk (12 %; [25]), Sagy et al. (7,6 %; [20]) und Giorgi et al. (6 %; [3]), keine diesbezüglichen Therapieabbrüche gab es bei Safakish [19], Chaves et al. [2], Yassin et al. [2] und Habib et al. [5]. In unserer Untersuchung gab es nur 2 nebenwirkungsbedingte Therapieabbrüche (2,3 %) während der insgesamt gesehen relativ kurzen Therapiedauer von knapp 2 Wochen. Entzugssymptome nach Abbruch der Therapie wurden nur bei Mazza et al. bei 2 entsprechend 6 % der Patienten berichtet.

## Limitationen

Die Aussagekraft der vorliegenden retrospektiven Auswertung ist vorsichtig zu bewerten, auch wenn hiermit erste Daten zur Versorgungsforschung im stationären Setting vorgelegt werden können. Sie umfasste einen relativ kurzen Beobachtungszeitraum von durchschnittlich 14 Tagen an einem einzigen Zentrum. Alle Patienten absolvierten ein weitgehend standardisiertes Therapieprogramm, das letztlich zeitgleich mit der THC-Applikation seinen Einfluss auf die Patienten ausübte. In unserer Studie erfolgte keine systematische Erfassung von Nebenwirkungen, ebenso wenig kamen FMS-typische Verlaufsfragebögen (z. B. FIQR) zur Anwendung. Es konnten nicht alle Patienten in der Kürze der stationären Aufenthaltsdauer auf die individuell optimale Dosis hochdosiert werden, da die Aufdosierung behutsam unter Berücksichtigung der Nebenwirkungen erfolgte.

## Fazit für die Praxis

Der genaue Wirkmechanismus von MC bei FMS-Patienten ist nicht geklärt. Die Ergebnisse der vorliegenden Auswertung weisen aber ähnlich wie in anderen Studien darauf hin, das THC eine medikamentöse Alternative in Ergänzung zu den bisher in verschiedenen Leitlinien empfohlenen Substanzen sein könnte. Vor einer Empfehlung zum klinischen Einsatz sind allerdings weitere Studien, vor allem zur Verträglichkeit und zur Effektivität bei mittel- und langfristiger Anwendung, notwendig. Dabei sind auch Risiken wie ein nichtmedizinischer Gebrauch bei Patienten mit psychiatrischen Nebendiagnosen zu beachten. Ebenso sollten die geeignete Applikationsform und das optimale THC/CBD-Verhältnis bei verschiedenen Schmerzerkrankungen als Studienziele im Fokus stehen.

## Footnotes

1 DIMDI: Deutsches Institut für Medizinische Dokumentation und Information, von 1969 bis 2020 Behörde des Bundesministeriums für Gesundheit, Herausgeber des Operationen- und Prozedurenschlüssels (OPS) in seiner jährlichen Fassung.
